# Reassessment of the Species in the *Euwallacea Fornicatus* (Coleoptera: Curculionidae: Scolytinae) Complex after the Rediscovery of the “Lost” Type Specimen

**DOI:** 10.3390/insects10090261

**Published:** 2019-08-22

**Authors:** Sarah M. Smith, Demian F. Gomez, Roger A. Beaver, Jiri Hulcr, Anthony I. Cognato

**Affiliations:** 1 Department of Entomology, Michigan State University, East Lansing, MI 48824, USA; 2 School of Forest Resources and Conservation, University of Florida, 136 Newins-Ziegler Hall, Gainesville, FL 32611, USA; 3 161/2 Mu 5, Soi Wat Pranon, T. Donkaew, A. Maerim, Chiangmai 50180, Thailand

**Keywords:** ambrosia beetle, invasive species, taxonomy, species delineation

## Abstract

Ambrosia beetles of the *Euwallacea fornicatus* (Eichhoff, 1868) species complex are emerging tree pests, responsible for significant damage to orchards and ecosystems around the world. The species complex comprises seven described species, all of which are nearly identical. Given that the morphology-defined species boundaries have been ambiguous, historically, there has been much disagreement on species validity, which was compounded by the presumed loss of the type series of *E. fornicatus*. The species complex was recently reviewed using morphometrics to associate the type specimens to the clades delineated with molecular data under the assumption of the lost type series. We rediscovered a syntype of *Xyleborus fornicatus*, and reevaluated the species in the complex using morphometrics. We propose the following taxonomic changes to the species complex: *Euwallacea fornicatus* (=*E. tapatapaoensis* (Schedl, 1951); = *E. whitfordiodendrus* (Schedl, 1942)) **syn. res.**); *E. fornicatior* (Eggers, 1923) (=*E*. *schultzei* (Schedl, 1951) **syn. nov.**); *E. kuroshio* (Gomez and Hulcr, 2018) and *E. perbrevis* (Schedl, 1951) **stat. res**. These taxonomic changes shift the species name associated with the widely used common names for two taxa, namely: *Euwallacea fornicatus* should be used for the “Polyphagous Shot Hole Borer”, and *E. perbrevis* for the “Tea Shot Hole Borer clade a”. A lectotype is designated for *X. fornicatus* in order to stabilize the use of the name.

## 1. Introduction

Xyleborine ambrosia beetles (Coleoptera: Curculionidae: Scolytinae: Xyleborini) are among the most damaging and invasive organisms, with impacts ranging from economic to ecological. Ecological and biological features, such as fungus-farming, haplodiploidy, and a wide host range, make the Xyleborini one of the most successful colonizers [1]. With ~1160 recognized species, this is the most species-rich tribe of Scolytinae, including some of the most widespread species [2].

The *Euwallacea fornicatus* species complex comprises seven species described from Asia and Oceania, namely: *E. fornicatus* (Eichhoff, 1868) (Sri Lanka), *E. fornicatior* (Eggers, 1923) (Sri Lanka), *E. whitfordiodendrus* (Schedl, 1942) (Malaysia), *E*. *schultzei* (Schedl, 1951) (Philippines), *E. perbrevis* (Schedl, 1951b) (Philippines), *E. tapatapaoensis* (Schedl, 1951) (Samoa), and *E. kuroshio* Gomez and Hulcr, 2018 (Japan). This species complex is arguably one of the most challenging in scolytine systematics, because the species are nearly identical. Given the blurred species boundaries, scolytine taxonomists disagreed on the validity of these species (see Stouthamer et al. [3] for a detailed synopsis). Wood [4] considered *E*. *schultzei*, *E. perbrevis*, *E. tapatapaoensis*, and *E. whitfordiodendrus* to be synonyms of *E. fornicatus*, and later, Wood and Bright [5] placed *E. fornicatior* in synonymy, leaving only *E. fornicatus* as valid.

*Xyleborus fornicatus* was described from an unknown number of specimens from Sri Lanka [6]. Eichhoff’s collection and types were deposited in the Zoological Museum in Hamburg, Germany (UZHM), and were destroyed when the museum was bombed during World War II. Approximately a dozen Eichhoff types were saved by K.E. Schedl, and now reside at the Naturhistorisches Museum Wien (NHMW) [5] (p. 3). Given that the type specimens were not present in either UZHM or NHMW, the type series was listed as ”lost” by Wood and Bright [5] in their catalog of bark and ambrosia beetles. However, a syntype was listed in a checklist of scolytine and platypodine types in the Zoological Museum, Museum and Institute of Zoology, Polish Academy of Science, Warsaw, Poland (MIIZ) [7], but this checklist was not listed in the catalog [5], and was subsequently largely ignored. This is one of several errors pertaining to type specimen deposition listed in Wood and Bright’s [5] catalog (see [8,9]).

Beginning in the 1980′s populations of what was presumed to be *Euwallacea fornicatus* became established outside Asia in Central and North America, Israel, and South Africa [4,10,11,12,13]. Stouthamer et al. [3] sampled *E. fornicatus s.l.* from multiple localities across Asia, as well as from introduced populations around the world. Their molecular phylogeny revealed that *E. fornicatus* was a complex consisting of three clades, presumed species, which were termed tea shot hole borer (TSHB), Kuroshio shot hole borer (KSHB), and polyphagous shot hole borer (PSHB). Gomez et al. [14] expanded the Stouthamer et al. [3] dataset and recognized the following four species: KSHB and PSHB, tea shot hole borer a (TSHBa), and tea shot hole borer b (TSHBb). These clade names are frequently used in the literature, but they do not have official taxonomic status and they are not officially recognized by the Entomological Society of America committee of common names. Gomez et al. [14] delineated species in the complex using morphometrics to associate molecular data with type specimens. With the assumption that the type series of *E. fornicatus* was lost [5], Gomez et al. [14] based their species concept on the specimens sequenced from the type locality of Sri Lanka, which corresponded to TSHBa clade.

**Figure 1:**
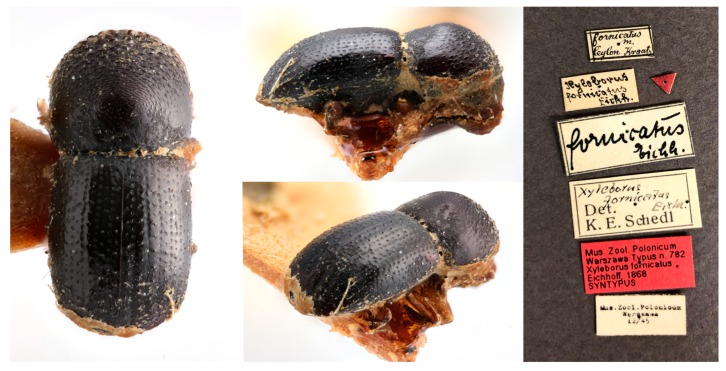
Syntype of *Xyleborus fornicatus*, female, from left to right: dorsal view, lateral view, posterior oblique view of declivity, and label information. Body length of 2.62 mm.

In this review, we examined the rediscovered *Xyleborus fornicatus* syntype (Figure 1) and additional non-type specimens of individuals in the *Euwallacea fornicatus* complex so as to revise diagnoses and provide new and revised distribution information.

## 2. Materials and Methods

We follow the clade names for the *Euwallacea fornicatus* species complex proposed by Stouthamer et al. [3], and those subsequently followed by Gomez et al. [14]. The syntype of *Xyleborus fornicatus*, four additional DNA vouchers of PSHB from Vietnam and Hong Kong, and two specimens from Vietnam, were measured and added into the mitochondrial cytochrome oxidase I (COI) dataset of Gomez et al. [14] (Table 1). Elytral and pronotal measurements followed the methodology of Gomez et al. [14], and were measured on the lateral view of the specimen. The elytral length was diagonally measured from the elytral base to the apex, and the pronotal length was diagonally measured from pronotal apex to the pronotal base. The pronotal width was measured at the widest point of the pronotum, and the elytra width equals the widest point of one elytron. We conducted a classification and regression tree (CART; rpart {rpart}) following Gomez et al. [14], with 94 individuals to corroborate relevant morphometric characters for clade discrimination. We used the CART model to predict the clade membership of the synonymized type specimens and the syntype of *Xyleborus fornicatus*. Statistical analyses were performed using R v.3.4.2 [15].

The syntype was photographed using an Olympus SZX16 stereomicroscope; each image is a composite of up to 50 separate images taken with a Canon EOS Rebel T3i camera, and later stacked using the Helicon Focus software (v 6.0, Helicon Soft, Kharkov, Ukraine). 

Additional museum specimens were also examined for distribution and host records. New distribution records are denoted with an asterisk. Because of the morphometric and protibial denticle number overlap between species, which can make species identification uncertain, the distribution data is divided into two parts, namely: records confirmed by DNA genotyping, and records based solely on morphology. The collections and codes are given below:

- California Academy of Sciences (CASC), San Francisco, California
- Zoological Museum, Museum and Institute of Zoology (MIIZ), Polish Academy of Science, Warsaw
- Michigan State University Arthropod Research Collection (MSUC), East Lansing, Michigan
- Natural History Museum (NHMB), Basel
- National Museum of Natural History (NMNH), Washington, D.C.
- Roger A. Beaver collection (RABC), Chiang Mai
- Robert J. Rabaglia collection (RJRC), Annapolis, Maryland

Species of the *Euwallacea fornicatus* species complex are known to attack over 412 species of plants in 75 families [16], but because of the taxonomic confusion regarding species limits, most of these records cannot be definitely associated with a particular species. The host records that can be definitively associated with species are listed below.

## 3. Results and Discussion

**Figure 2:**
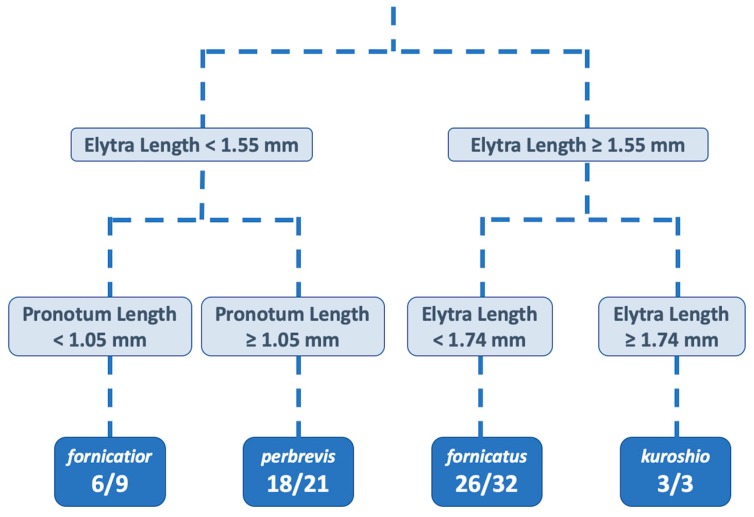
Classification tree from the classification and regression tree (CART) analysis built on 65 individuals representing the four species within the *E. fornicatus* complex. The relevant characters for the node splits are shown. The classification rates are expressed as the number of correct classifications divided by the number of observations (individual beetles) in the node for the individuals used to train the CART model.

Additional vouchers were added to the original dataset from Gomez et al. [14] (Table 1), with no changes in the CART model previously proposed. The CART analysis classified the specimens into the four phylogenetic clades (Figure 2). The classification accuracy for the training data (*n* = 65) was 82%. The classification accuracy of the remaining individuals (*n* = 29) was 76%. The obtained classification tree supports the findings in Gomez et al. [14], where the morphotype identity was primarily explained by the elytra length and the pronotum length. The previously synonymized types and the re-discovered *X. fornicatus* syntype were classified with the CART model into the pre-established clades. The types of synonymized species were classified consistently with Gomez et al. [14]. The *X. fornicatus* syntype was classified as the PSHB clade. The comparative measurements for the four species based on the original dataset in Gomez et al. [14], and the additionally measured specimens (by SMS and RAB), are described in Table 2. Based on the phylogenetic data, CART analysis, and location, we suggest the following taxonomic changes.

### 3.1. Taxonomy

#### 3.1.1. Euwallacea Fornicatior (Eggers)

*Xyleborus fornicatior* Eggers, 1923

*Xyleborus fornicatus fornicatior* Eggers, 1923: [17]

*Euwallacea fornicatior* (Eggers): [5] (as a synonym of *E. fornicatus*)

*Xyleborus schultzei* Schedl, 1951. **syn. nov.**

**Diagnosis.** Specimens of this species are stouter than those of *E. perbrevis*, with an elytra length of 1.40–1.46 mm and a pronotum length of 1.02–1.06 mm, with six or seven socketed denticles on the margin of the protibia. The pronotum width in this clade is 1–1.06 mm and the elytra width is 0.48–0.52 mm (Table 2). The species can also be distinguished by its more strongly convex elytral disc, giving it a humped appearance.

**Types.** Holotype *Xyleborus fornicatior*: Ceylon (Sri Lanka) (NMNH); lectotype *Xyleborus schultzei*: Philippines (NHMW).

**Clades.** TSHB clade 1a [3] and TSHBb [14].

**New records.** CHINA: NW Sichuan, Guanxian, 103.36[E], 31.00[N], 700 m, 8–20.vii.1990, J. Kolibač (RABC, 1). [Federated States of Micronesia]: Ponape I., U. Dist., Awakpa, 2.iii.1948, H.S. Dybas, ex under bark of breadfruit (NMNH, 1). INDIA: [Assam], Darjeeling D[istr.], Rally Khola, 600 m, 9.x.1981, B. Bhakta (RABC, 1); as previous except: Chuba, 1000 m, 27‒28.iii.1983 (NHMB, 1). INDONESIA: Sulawesi Utara, Dumoga-Bone N.P., ca 200 m, lowland forest, malaise trap, xii.1985 (RABC, 1). [MALAYSIA]: Sarawak, Bako NP, 50 m, branch, 29.x.‒2.xi.1998, B. Jordal (RABC, 1). TAIWAN: Ping Tung co., Maolin Natl Scenic Area, 30.x.2011, R. A. Beaver (RABC, 1). THAILAND: Nakhon Sri Thammarat, in durian plantation, ex durian branch, W. Sittichaya, 1.ii.2010 (RABC, 1); as previous except: 1.iii.2010 (RABC, 1). Trang, Khao Chong Nature Educ. Centre, 7.35N, 99.46E, Lowland Trop. Rain. For. 21–24.vii.1996, R. A. Beaver (RABC, 1).

Distribution (molecular data). Papua New Guinea, Singapore. 

**Distribution (morphological data).** China * (Sichuan), Federated States of Micronesia, India (Assam *, Kerala, Tamil Nadu), Indonesia (Java, Sulawesi *), East * and West Malaysia, Papua New Guinea, Philippines, Singapore, Sri Lanka, Taiwan *, and Thailand *.

**Host plants.** Recorded from tea (*Camellia sinensis*) (Theaceae), *Albizzia* and *Tephrosia* (Leguminosae) [17,18,19], durian (*Durio zibethinus)* (Malvaceae), and breadfruit (*Artocarpus altilis*) (Moraceae).

**Remarks.** The types of *E. fornicatior* and *E. schultzei* were classified as belonging to the TSHBb clade based on body measurements. Gomez et al. [14] classified *E. schultzei* as belonging to TSHBb, but the species was retained as a synonym of *E. fornicatus*. The types were directly compared and were found to be conspecific. Given the results of the Gomez et al. [14] analysis, and the overall similarity of the type specimens, *E. schultzei* is here placed in synonymy with *E. fornicatior*.

#### 3.1.2. Euwallacea fornicatus (Eichhoff)

*Xyleborus fornicatus* Eichhoff, 1868

*Xyleborus fornicatus fornicatus* Eichhoff, 1868: [17]

*Euwallacea fornicatus* (Eichhoff): [4]

*Xyleborus tapatapaoensis* Schedl, 1951: [4]

*Xyleborus whitfordiodendrus* Schedl, 1942. **syn. res.**

**Diagnosis.** Specimens of this species have an elytra length of 1.44–1.72 mm and a pronotum length of 1.02–1.16 mm, with eight or nine socketed denticles on the margin of protibia. The pronotum width in this clade is 1–1.14 mm and the elytra width is 0.48–0.62 mm (Table 2).

**Types.** Lectotype (designated below) *Xyleborus fornicatus*: Ceylon (Sri Lanka) (MIIZ); lectotype *Xyleborus tapatapaoensis* Samoa (NHMW); lectotype *Xyleborus whitfordiodendrus*: Malaysia (NHMW).

**Clade.** PSHB [3,4].

**New records.** CHINA: Chongqing, Gele Mtn, 5.v.2016, Tian-Shang, Lv-Jia (RABC, 1). Yunnan, Xishuangbanna, Sanchahe Nat. Res., N 22°09.784′ E 100°52.256′, 2186 m, 29‒30.v.2008, A.I. Cognato (MSUC, 1). INDIA: S. India, coffee research station, K. Gopinath, 17.ii. [19] 60, 13753, ex *Erythrina lithosperma* (NMNH, 2). Uttar Pradesh, Dehra Dun, Ladpur, 686 m, 20.x.1923, B.D. Saklani, F.C. Hadden, ex *Ricinus communis* (CASC, 7); as previous except: Asarorie, 704 m, 26.xi.1932 (CASC, 1); [West] Bengal, Samsingh, 1800 [m], Kalimpong, 8.x. [19] 33, ex unknown wood (NMNH, 3). JAPAN: Bonin Islands, Chichi-jima, Omu-ra, 6.vii.1949, A.R. Mead, ex schoolhouse under bark (NMNH, 1). MALAYSIA: Sabah, Sipitang, Mendolong, 4.v.1988, S. Adebratt (RABC, 1). SRI LANKA: Bad. [=Badulla] Dist. Haputale, 4.vi.1973, S.L. Wood, ex *Ficus* sp. (NMNH, 9); Kan. [=Kandy] Dist. Laksapana, 1200′, 23‒29.ix.1970, O.S. Flint, Jr (NMNH, 1). VIETNAM: Cao Bang prov., 22°34.5′ N 105°52.4′ E, ~1080 m, 14.iv.2014, VN28, Cognato, Smith, Pham, ex *Cunninghamia* branches (MSUC, 4). Dong Nai prov., Cat Tien NP, 11.46050, 107.37375, 379 m, 22.ii.2017, VN93, A.I. Cognato, T.A. Hoang, ex 4−5 cm dia branches (MSUC, 84). Ninh Binh prov., Cuc Phuong N.P., 5.iii.2018, 20.33296, 105.61259, 279 m, A.I. Cognato and S.M. Smith, VN 110, ex 4−6 cm diameter branch (MSUC, 25). Thua Thien-Hue prov., Bach Ma NP, 16.18902, 107.8498, 1193 m, 15.ii.2017, VN54, A.I. Cognato, T.A. Hoang, ex 1−4 cm dia branches (MSUC, 2); as previous except: 16.22897, 107.85349, 415 m, 15.ii.2017, VN60, ex 4 cm dia branches (MSUC, 1). Tuyen Quang prov., Doi Can Tuyen Quang, N21.72740 E105.22742, 15.iv.2015, R.J. Rabaglia, ex funnel trap (RJRC, 2). Yen Bai prov., Tan Huong, 21.82410 104.89651, 15.iv.2015, R.J. Rabaglia, ex funnel trap (RJRC, 1).

**Distribution (molecular data).** China (Guizhou, Hong Kong), Japan (Okinawa), Thailand, and Vietnam, and introduced into Israel, South Africa, and the United States (California).

**Distribution (morphological data).** China (Chongqing, Guizhou, Hong Kong, and Yunnan), India (Uttar Pradesh), Japan (Bonin Islands and Okinawa), Malaysia (Sabah) *, Samoa, Sri Lanka, Taiwan, Thailand, and Vietnam. This species has been introduced into Israel, South Africa, and the United States (California; cited as PSHB and/or *E. whitfordiodendrus*). Distribution records published prior to 2018 may not reflect the actual species distribution.

**Host plants.** It is here recorded from two genera: *Cunninghamia* (Cupressaceae) and *Erythrina* (Fabaceae). In Samoa, it has been recorded as *Xyleborus tapatapoensis* from *Albizia* sp., *Bauhinia variegata, Erythrina orientalis* (Fabaceae), *Ochroma lagopus* (Malvaceae), and *Milicia* (=*Chlorophora*) *excelsa* (Moraceae) [20]. It has also been recorded from the following hosts under different names: *Callerya* (Fabaceae), *Persea americana* (Lauraceae) ([21,22] as *Euwallacea* sp. #1 and PSHB, respectively), *Robinia* (Fabaceae) [23], *Sambucus* (Adoxaceae), *Liquidambar* (Altingiaceae), *Schinus* (Anacardiaceae), *Alnus* (Betulaceae), *Ricinus* (Euphorbiaceae), *Acacia* (Fabaceae), *Carya*, *Quercus* (Fagaceae), *Juglans* (Juglandaceae), *Umbellaria* (Lauraceae), *Magnolia* (Magnoliaceae), *Ficus*, *Morus* (Moraceae), *Eucalyptus* (Myrtaceae), *Fraxinus* (Oleaceae), *Platanus* (Platanaceae), *Prunus* (Rosaceae), *Populus*, *Salix* (Salicaceae), *Acer* (Sapindaceae), *Ailanthus* (Simaroubaceae), and *Ulmus* (Ulmaceae) ([22,24] as PSHB).

**Remarks**. The syntype of *X. fornicatus*, as well as the types of *X. tapatapaoensis* and *X. whitfordiodendrus*, were directly compared, classified as belonging to the PSHB clade based on body measurements and protibal denticle number, and found to be conspecific. Gomez et al. [14] considered the clade of PSHB to be *E. whitfordiodendrus*, and their analyses indicated that *E. tapatapaoensis* fit within the PSHB clade, but the species was never placed in synonymy with *E. whitfordiodendrus* and was retained as a synonym of *E. fornicatus*. Both species had been previously considered synonyms of *E. fornicatus* [4], and are again recognized as such.

To ensure correct and consistent application of the name, here, we designate the female syntype as the lectotype of *Xyleborus fornicatus* Eichhoff. The lectotype is labeled “*fornicatus* m. Ceylon Kraatz [handwritten]\*Xyleborus fornicatus* Eichh. [handwritten]\[red triangle]\*fornicatus* Eichh. [handwritten]\*Xyleborus fornicatus* Eichh. [handwritten] Det. K.E. Schedl [typed]\Mus. Zool. Polonicum Warszawa 12/45 [typed]\[red label] Mus. Zool. Polonicum Warszawa Typus 782 *Xyleborus fornicatus* Eichhoff, 1868 SYNTYPUS\[red label] LECTOTYPE *Xyleborus fornicatus* Eichhoff [typed]”.

#### 3.1.3. *Euwallacea kuroshio* Gomez and Hulcr

*Euwallacea kuroshio* Gomez and Hulcr, 2018 [14].

**Diagnosis.** The specimens of this species have an elytra length of 1.50–1.82 mm and a pronotum length of 1.08–1.16 mm, with 8 to 11 socketed denticles in the protibiae. The pronotum width in this species is 1.06–1.16 mm and the elytra width is 0.52–0.56 mm (Table 2).

**Holotype.** Japan (NMNH).

**Clade.** KSHB [3,14].

New records. None.

**Distribution (molecular data).** Indonesia (East Java), Japan (Okinawa), and Taiwan. This species has been introduced into Mexico and the United States (California) [3,14,25].

Distribution (morphological data). Same as for molecular data.

**Host plants.** This species is known from *Sambucus* (Adoxaceae), *Liquidambar* (Altingiaceae), *Schinus*, *Searsia* (Anacardiaceae), *Ambrosia*, *Baccharis* (Asteraceae), *Alnus* (Betulaceae), *Ricinus* (Euphorbiaceae), *Quercus* (Fagaceae), *Cassia*, *Persea* (Lauraceae), *Ficus* (Moraceae), *Eucalyptus* (Myrtaceae), *Juglans*, *Pterocarya* (Juglandaceae), *Magnolia* (Magnoliaceae), *Fraxinus* (Oleaceae), *Platanus* (Platanaceae), *Populus*, *Salix* (Salicaceae), *Nicotiana* (Solanaceae), and *Tamarix* (Tamaricaceae) [24,26,27].

#### 3.1.4. *Euwallacea perbrevis* (Schedl) Stat. Res.

*Xyleborus perbrevis* Schedl, 1951

*Euwallacea perbrevis* (Schedl): [4] (as a synonym of E. fornicatus)

**Diagnosis.** Specimens of this species have elytral length of 1.42–1.68 mm and a pronotum length of 1.04–1.16 mm, with 7 to 10 socketed denticles on the edge of the protibia. The pronotum width in this clade is 1.02–1.14 mm and the elytra width is 0.48–0.56 mm (Table 2).

**Type.** Holotype *Xyleborus perbrevis*: Philippines (NHMW).

**Clade.** TSHB clade 1b [3] and TSHBa [14].

**New records.** AMERICAN SAMOA: Tutuila Isl., Malaeimi, 14.31725 [S], 170.73653 [W], paramethanol baited multifunnel trap in ACE lumberyard, 19‒26.viii.2016, M. Schmaedick (RABC, 8). BRUNEI: Temburong, Sg. Babi mouth, 4°32.5′N, 115°11′E, 80 m, ex liane, 24.ii.1992, R. A. Beaver (RABC, 3). CHINA: Hainan, Wu Zhi Shan City, 18.902°N, 109.663°E, 703 m, 2.xii.2016, Tian-Shang, Lv-Jia (RABC, 1); as previous except: Jianfengling Nat. For. Park, 18.700°N, 108.811°E, 133m, 4.xii.2016 (RABC, 3); as previous except: Bawangling Nat. For. Park, 19.117°N, 109.080°E, 119 m, 5.xii.2016 (RABC, 1). FIJI: Viti Levu, Galoa, ex *Xylopia pacifica*, 17‒18.x.1988, R. A. Beaver (RABC, 1); as previous except: Mt. Korobaba, 18.v.1985 (RABC, 2); as previous except: Namosi Rd, 6 km., 26.xii.1982, 3.iv.1983 (RABC, 2); as previous except: Savura Ck, ex *Cyathocalyx* sp., 28.viii.1982, 15.x.1982 (RABC, 2); as previous except: ex *Myristica castaneifolia*, 1.viii.1983 (RABC, 1); as previous except: ex *Casearia disticha*, 12.iii.1983 (RABC, 1); as previous except: Suva, ex *Artocarpus altilis*, 20.xii.1982 (RABC, 1); as previous except: Wailoku, ex *Trichospermum* sp., 31.xii.1982 (RABC, 1). JAPAN: Okinawa I., Miyako, 28.ix.2005, H. Kajimura (RABC, 1). [INDONESIA]: Java, Bogor, viii.1964, H.L.H. Krauss, ex *Mangifera indica* (NMNH, 1). MALAYSIA: Perak, Kuala Kangor, x.1964, N.L.H.K., ex *Theobroma cacao* (NMNH, 1). Sabah, nr. Tawau, ex *Acacia mangium*, iv.2012, M. J. Wingfield (RABC, 1). Selangor, Sendana, ix.1964, N.L.H. Krauss, ex *Mangifera indica* (NMNH, 2); as previous except: ex *Theobroma cacao* (NMNH, 4); as previous except: ex ponnelo [sic! = pomelo] (NMNH, 1). PALAU: Peleliu I., 29.i.1948, H.S, Dybas, ex ridge under bark from dead tree (NMNH, 1). PANAMA: Canal Zone, Barro Colorado Island, vi–viii.1979, G.C. Stevens, ex reared from sapwood of *Bursera simarouba* (NMNH, 1). PHILIPPINES: Mindanas, Davao, v.1964, N.L.H. Krauss, ex *Theobroma cacao* (NMNH, 6). SRI LANKA: Talawakelle, St. Coombs [Estate], [Tea Research Institute], 1928, ex in tea stems (NMNH, 15). TIMOR LESTE: Emera, Railaco, Lihu, Kamalbun, S 8.67935, E 125.41665, ex *Theobroma cacao*, 20.ii.2019, T. Popic (RABC, 3). UNITED STATES: Hawaii, Oahu, Ewa Coral Plain, vii.1919, J.C. Bridwell, ex *Erythrina* (NMNH, 8); Wahiawa, xi.[19]54, Ford, ex *Aleurites* (NMNH, 1). RÉUNION: Bras Madeline, 12.vi.1996, S. Quilici, ex sur *Litchi sinensis* (NMNH, 1). SINGAPORE: Mandai mangrove, 29.iii.[19]88, D.H. Murphy, ex upper branches of a fallen *Avic*. [= *Avicennia*] *alba* (NMNH, 3). VIETNAM: Ninh Binh prov., Cuc Phuong N.P., 5.iii.2018, 20.34932, 105.59669, 431 m, A.I. Cognato and S.M. Smith, VN 113b, ex *Terminalia myriocarpa*; large tree fall trunk, 8 cm (MSUC, 1).

**Distribution (molecular data).** American Samoa, Australia, China (Hainan), India, Indonesia (Java), Papua New Guinea, Sri Lanka, and Thailand, and introduced into the United States (Florida and Hawaii) [3,14,21].

**Distribution (morphological data).** This species is confirmed from American Samoa, Australia, Brunei *, China * (Hainan), Fiji *, India, Indonesia (Java), Japan (Okinawa) *, Malaysia * (Java, Sabah), Palau *, Papua New Guinea, Philippines, Réunion *, Singapore *, Sri Lanka, Taiwan, Thailand, Timor Leste *, and Vietnam *, and introduced into the United States (Florida and Hawaii) [14], Costa Rica, and Panama ([28], reported as *E. fornicatus*).

**Host plants.** The species is here recorded from 16 genera in 13 families: *Avicennia* (Acanthaceae), *Mangifera* (Anacardiaceae), *Cyathocalyx*, *Xylopia* (Annonaceae), *Bursera* (Burseraceae), *Terminalia* (Combretaceae), *Aleurites* (Euphorbiaceae), *Acacia*, *Erythrina* (Fabaceae), *Theobroma* and *Trichospermum* (Malvaceae), *Artocarpus* (Moraceae), *Myristica* (Myristicaceae), *Citrus* (Rutaceae), *Casearia* (Salicaceae), and *Litchi* (Sapindaceae). It has been recorded from the following hosts under different names: *Protium* (Burseraceae), *Cedrela* (Meliaceae), *Brosimum* (Moraceae) ([28] as *E. fornicatus*), *Camellia sinensis* (Theaceae) ([21] as *Euwallacea* sp. #4), *Annona* (Annonaceae), *Bursera* (Burseraceae), and *Albizia*, *Lysiloma* (Fabaceae) ([29] as *Euwallacea* nr. *fornicatus*).

**Remarks**. *Euwallacea perbrevis* was previously considered a synonym of *E. whitfordiodendrus* by Gomez et al. [14], despite being classified by the CART analysis as belonging to TSHBa, *E. fornicatus*. Given the rediscovery of the *E. fornicatus* syntype and its placement in the CART analysis as PSHB, the holotype of *E. perbrevis* remained the only type in the species group with morphology congruent to the TSHBa clade, and is here recognized as valid.

### 3.2. Identity of the Tea Shot Hole Borer

“*Euwallacea fornicatus*” in Sri Lanka was first referred to as the “tea shot hole borer” by Speyer [30], and was extensively used in the literature in publications regarding biology and control (e.g., [31,32]). In his paper proposing the common name, Speyer [30] did not comment on the species’ appearance. Later, several authors noted differences in the host preference among the specimens, which correlated with the body size and elytral convexity, implying the occurrence of multiple species in Sri Lanka that differ in their preference for tea (e.g., [17,31,33]). *Euwallacea fornicatior*, *E. fornicatus,* and *E. perbrevis* all occur in Sri Lanka. Different authors give varying sizes for “*E. fornicatus*”; 2.20–2.35 by Beeson [17], 2.3 mm by Gadd and Loos [33], and ~2.5 mm by Walgama [32]. Thus, the studies imply different species being the tea shot hole borer. Beeson [17], and Gadd and Loos [33] suggest *E. fornicatior,* while Walgama [32] suggests *E. perbrevis*, which has an upper size limit of 2.5 mm (Table 2). O’Donnell et al. [21] sequenced specimens of “*Euwallacea* sp. #4’ from tea (*Camellia sinensis*) plantations in Sri Lanka. These COI sequences were subsequently used by Stouthamer et al. [3], and were found to belong to the TSHBa clade, now considered *E. perbrevis*. The species has also been historically associated with tea, as a large series of *Euwallacea perbrevis* specimens and their associated galleries were collected from tea at the Tea Research Institute in 1928 (NMNH; see above), also supporting this species as the tea pest. Given the size of the species, referenced by Walgama [32], and that O’Donnell et al. [21] only collected TSHBa individuals from tea, it is most likely that *E. perbrevis*, not *E. fornicatus,* is the tea shot hole borer.

## 4. Conclusions

The discovery of a lost syntype of *E. fornicatus* allowed us to reevaluate the species limits of this confusing species complex. Using a morphometric analysis, we redefined *E. fornicatus* and synonymized a species that fell within this group of morphologically similar species. All of the species with the complex are now validated by morphometric and phylogenetic analyses. Most importantly, we stabilized the common names with their association with valid species, thus facilitating communication among laypersons.

## Figures and Tables

**Table 1:** GenBank accession numbers for the 70 COI sequences used in this study, and the associated clade name, species, and locality details.

GenBank Accession Number	Clade	Species	Country	Locality
KU727004	KSHB	*E. kuroshio*	Japan	Okinawa
KU727005	KSHB	*E. kuroshio*	Japan	Okinawa
KU727008	KSHB	*E. kuroshio*	Taiwan	Not specified
MH276944	KSHB	*E. kuroshio*	Indonesia	East Java
MH276945	KSHB	*E. kuroshio*	Indonesia	East Java
MH276946	KSHB	*E. kuroshio*	USA	California
MH276947	KSHB	*E. kuroshio*	USA	California
MH276948	KSHB	*E. kuroshio*	USA	California
MH276949	KSHB	*E. kuroshio*	USA	California
MH276950	KSHB	*E. kuroshio*	USA	California
JX912724	PSHB	*E. fornicatus*	USA	California
KU727012	PSHB	*E. fornicatus*	Vietnam	Phu Yen
KU727014	PSHB	*E. fornicatus*	Vietnam	Gia Lai
KU727016	PSHB	*E. fornicatus*	Thailand	Chiang Mai
KU727019	PSHB	*E. fornicatus*	Vietnam	Yen Bai
KU727020	PSHB	*E. fornicatus*	Vietnam	Not specified
KU727021	PSHB	*E. fornicatus*	USA	California
KU727023	PSHB	*E. fornicatus*	USA	California
KU727026	PSHB	*E. fornicatus*	Vietnam	Yen Bai
KU727027	PSHB	*E. fornicatus*	Japan	Okinawa
MH276936	PSHB	*E. fornicatus*	China	Guizhou
MH276937	PSHB	*E. fornicatus*	China	Guizhou
MH276938	PSHB	*E. fornicatus*	Thailand	Phetchabun
MH276939	PSHB	*E. fornicatus*	Japan	Okinawa
MH276940	PSHB	*E. fornicatus*	Vietnam	Hoan Kiem
MH276941	PSHB	*E. fornicatus*	China	Hong Kong
MH276942	PSHB	*E. fornicatus*	USA	California
MH276943	PSHB	*E. fornicatus*	Thailand	Chiang Mai
MN266860	PSHB	*E. fornicatus*	China	Hong Kong
MN266859	PSHB	*E. fornicatus*	Vietnam	Thua Thien-Hue
MN266861	PSHB	*E. fornicatus*	Vietnam	Ninh Binh
MN266858	PSHB	*E. fornicatus*	Vietnam	Cao Bang
KU726992	TSHBa	*E. perbrevis*	Australia	Sunshine Coast
KU726995	TSHBa	*E. perbrevis*	Thailand	Not specified
KU726996	TSHBa	*E. perbrevis*	USA	Florida
KU726997	TSHBa	*E. perbrevis*	Thailand	Not specified
KU726998	TSHBa	*E. perbrevis*	Thailand	Not specified
KU726999	TSHBa	*E. perbrevis*	Thailand	Chiang Mai
KU727001	TSHBa	*E. perbrevis*	Thailand	Chumphon
KU727003	TSHBa	*E. perbrevis*	Thailand	Surat Thani
MH276907	TSHBa	*E. perbrevis*	Indonesia	East Java
MH276908	TSHBa	*E. perbrevis*	Indonesia	East Java
MH276909	TSHBa	*E. perbrevis*	Indonesia	East Java
MH276910	TSHBa	*E. perbrevis*	Indonesia	East Java
MH276911	TSHBa	*E. perbrevis*	Indonesia	East Java
MH276912	TSHBa	*E. perbrevis*	Indonesia	East Java
MH276913	TSHBa	*E. perbrevis*	Papua New Guinea	Eastern Highlands
MH276914	TSHBa	*E. perbrevis*	USA	Florida
MH276915	TSHBa	*E. perbrevis*	USA	Florida
MH276916	TSHBa	*E. perbrevis*	USA	Florida
MH276917	TSHBa	*E. perbrevis*	Thailand	Nakhon Si Thammarat
MH276918	TSHBa	*E. perbrevis*	Thailand	Nakhon Si Thammarat
MH276919	TSHBa	*E. perbrevis*	Thailand	Nakhon Si Thammarat
MH276920	TSHBa	*E. perbrevis*	Thailand	Trat
MH276921	TSHBa	*E. perbrevis*	Thailand	Trat
MH276922	TSHBa	*E. perbrevis*	Thailand	Chanthaburi
MH276923	TSHBa	*E. perbrevis*	Thailand	Chanthaburi
MH276924	TSHBa	*E. perbrevis*	Thailand	Trat
MH276925	TSHBa	*E. perbrevis*	China	Hainan
MH276926	TSHBa	*E. perbrevis*	Thailand	Chiang Mai
MH276927	TSHBa	*E. perbrevis*	Thailand	Chiang Mai
MH276928	TSHBa	*E. perbrevis*	American Samoa	Aoa
MH276929	TSHBa	*E. perbrevis*	Papua New Guinea	Madang
KU726991	TSHBb	*E. fornicatior*	Singapore	Not specified
MH276930	TSHBb	*E. fornicatior*	Papua New Guinea	Madang
MH276931	TSHBb	*E. fornicatior*	Papua New Guinea	Madang
MH276932	TSHBb	*E. fornicatior*	Papua New Guinea	Madang
MH276933	TSHBb	*E. fornicatior*	Papua New Guinea	Oro
MH276934	TSHBb	*E. fornicatior*	Papua New Guinea	Oro
MH276935	TSHBb	*E. fornicatior*	Papua New Guinea	Oro

**Table 2:** Comparative table of measurements (mm) for *Euwallacea fornicatior*, *E. fornicatus*, *E. kuroshio*, and *E. perbrevis*. Measurements for the total length, pronotal and elytral width, and length/width ratios are measured in the dorsal view, while the pronotal and elytral length are measured in a lateral view on a diagonal, as detailed by Gomez et al. [14].

Species	Total Length (Dorsal)	Length/Width Ratio (Dorsal)	Elytral Length (Lateral; Diagonal)	Pronotal Length (Lateral; Diagonal)	Elytral Width (Dorsal)	Pronotal Width (Dorsal)	Protibial Denticle Number
*fornicatior*	2.20–2.37	2.15−2.35	1.40–1.46	1.02–1.06	0.48–0.52	1.00–1.06	6–7
*fornicatus*	2.60−2.70	2.25−2.36	1.44–1.72	1.02–1.16	0.48–0.62	1.00–1.14	8–9
*kuroshio*	2.40−2.80	2.17−2.40	1.50–1.82	1.08–1.16	0.52–0.56	1.06–1.16	8–11
*perbrevis*	2.30−2.50	2.46−2.55	1.42–1.68	1.04–1.16	0.48–0.56	1.02–1.14	7–10
